# Relaxivity Modulation of Gd-HPDO3A-like Complexes by Introducing Polar and Protic Peripheral Groups

**DOI:** 10.3390/molecules29194663

**Published:** 2024-09-30

**Authors:** Sara Camorali, Loredana Leone, Laura Piscopo, Lorenzo Tei

**Affiliations:** Department of Science and Technological Innovation, Università del Piemonte Orientale, Viale T. Michel 11, 15121 Alessandria, Italy; sara.camorali@uniupo.it (S.C.); loredana.leone@uniupo.it (L.L.); laura.piscopo@uniupo.it (L.P.)

**Keywords:** Gd(III) complexes, relaxometry, macrocyclic chelators, functional groups

## Abstract

In the last three decades, high-relaxivity Magnetic Resonance Imaging (MRI) contrast agents (CAs) have been intensively sought, aiming at a reduction in the clinically injected dose while maintaining the safety of the CA and obtaining the same pathological information. Thus, four new Gd(III) complexes based on modified 10-(2-hydroxypropyl)-1,4,7,10-tetraazacyclododecane-1,4,7-triacetic acid (HP-DO3A) macrocyclic structure were designed and synthesized by introducing further polar and protic functional groups (amides, phosphonates, and diols) adjacent to the metal-coordinated hydroxyl group. A detailed ^1^H NMR relaxometric analysis allowed us to investigate the effect of these functional groups on the relaxivity, which showed a 20–60% increase (at 0.5 T, 298 K, and pH 7.4) with respect to that of clinically approved CAs. The contribution of the water molecules H-bonded to these peripheral functional groups on the relaxivity was evaluated in terms of the second sphere effect or prototropic exchange of labile protons.

## 1. Introduction

**Figure 1:**
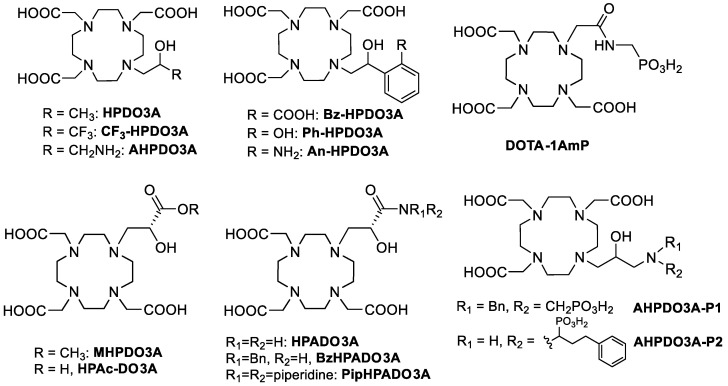
HPDO3A-like ligands discussed in the text.

Gd(III)-based contrast agents (GBCAs) are used in more than one-third of Magnetic Resonance Imaging (MRI) scans to improve the clarity of the images of the body’s internal structures where the agent is localized [1,2,3]. Gd(III) complexes are the most commonly used compounds for contrast enhancement in clinics, although other paramagnetic metal complexes such as Mn(II) and Fe(III) are currently under intense scrutiny for preclinical applications [4,5]. The efficiency of a GBCA is determined by its relaxivity, defined as the increase in the relaxation rate given by a 1 mM solution of the paramagnetic complex. Over the past three decades, various approaches have been employed to modulate and enhance relaxivity while consistently prioritizing the safety of the agent for its in vivo use, as indicated by its thermodynamic stability and kinetic inertness in physiological conditions [3]. Furthermore, to expand the MRI application into the molecular imaging approach, a specialized class of contrast agents known as bioresponsive GBCAs has been developed [6,7]. Nevertheless, to improve the relaxivity of GBCAs, research has focused on Gd(III) complexes featuring a faster water exchange rate [8], a higher number of coordinated water molecules (*q*) [9,10,11], lipophilic groups capable of interacting with serum proteins [12,13,14], or hydrophilic groups that can hydrogen-bond to a large number of second-sphere water molecules [15]. The renewed interest in the search for improved GBCAs that can be used at lower doses in vivo has brought to the approval of a new Gd(III) complex, Gadopiclenol, with a relaxivity three times higher than that of GBCAs currently employed in the clinics [16]. This agent combines the advantage of a *q* = 2 Gd(III) complex based on GdPCTA (3,6,9,15-tetraazabicyclo[9.3.1]pentadeca-1 (15),11,13-triene-3,6,9-triacetic acid) with the presence of a large number of hydrophilic groups that increase the second sphere water molecules contributing to the relaxivity. Another approach that has been recently pursued to improve the relaxivity of already clinically approved GBCAs was the modulation of one pendant arm of the chelating unit to introduce functional groups able to form strong H-bonds with second sphere water molecules and/or accelerate the exchange with the bulk water of the mobile protons present at a relatively short distance from the Gd(III) center [17]. The prototropic exchange effect is particularly effective in GdHPDO3A-like complexes (GdHPDO3A = Gadoteridol, a Bracco commercialized GBCA, Figure 1) where a hydroxyl group is directly coordinated to the paramagnetic center [18]. The exchange of the OH proton has been accelerated by the presence of adjacent protic functional groups such as amines [19], phenols [20], amides [21], carboxylic acids [22], or phosphonate groups [19] (Figure 1). In particular, GdHPADO3A-like complexes provide labile amide protons capable of establishing an impressive acid-catalyzed proton exchange process with the metal-coordinated OH group and second-sphere water molecules, causing a remarkable relaxivity increase at acidic pH (pH 4.2) more than double than that observed at pH 7.4 [21]. This effect was found to be dependent on the number of amide protons, being more pronounced for the primary amide derivative and less effective in the case of the tertiary amide. In another example, GdDOTA-1AmP (Figure 1), the relaxivity increase was ascribed to the presence of a water molecule, H-bonded to the NH proton and the phosphonate group, that experienced faster relaxation compared to other typical second-sphere water molecules [15].

**Figure 2:**
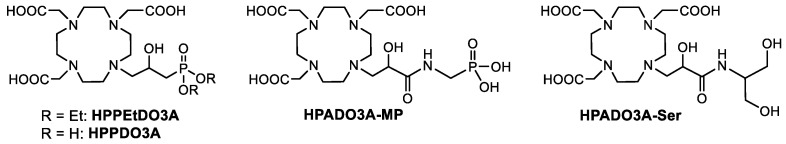
Novel HPDO3A-like ligands synthesized in this work.

Taking into account the structural features of the ligands reported in Figure 1, we have designed and synthesized a series of novel HPDO3A-based ligands bearing one or more polar and/or protic functional groups next to the coordinated hydroxyl group of HPDO3A (Figure 2). In particular, we designed two ligands bearing a phosphonate group linked through a simple methylene group (HPPDO3A) and through an amidic group (HPADO3A-MP). In the case of HPPDO3A, the correspondent ligand with the ethyl-protected phosphonate group (HPPEtDO3A) was synthesized in order to evaluate the effect of the free phosphonate on the relaxivity. Finally, another ligand combining the presence of a secondary amide and a hydrophilic dihydroxypropyl group (HPADO3A-Ser) was designed and synthesized. A ^1^H NMR relaxometric study on the correspondent Gd(III) complexes was conducted to investigate the contribution of the prototropic exchange and/or of the second sphere water molecules to the relaxivity.

## 2. Results and Discussion

**Scheme 1:**
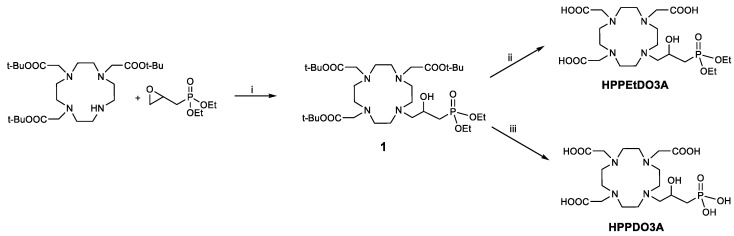
Synthesis of HPPEtDO3A and HPPDO3A. i: *t*BuOH, reflux, 18 h; ii: TFA, DCM 1:1, 18 h; iii: BrSi(CH_3_)_3_, DCM, 0 °C, 15 min, then RT, 18 h.

The synthesis of the ligands with a 2-hydroxypropylphosphonate pendant started from DO3A(OtBu)_3_ by reaction with diethyl (2-oxiranylmethyl)phosphonate (Scheme 1). This epoxide was obtained by exploiting a reported procedure by oxidation of diethyl allylphosphonate with m-chloroperbenzoic acid [23]. The intermediate **1** was purified by semi-preparative HPLC-MS, and then the *tert*-butyl groups were deprotected using trifluoroacetic acid to obtain the HPPEtDO3A ligand. On the other hand, to obtain the completely deprotected ligand, HPPDO3A, the McKenna reaction [24] was exploited, in which bromotrimethylsilane (BTMS) is used to deprotect the ethyl phosphonate esters. In our case, the *tert*-butyl esters were also deprotected by BMTS, and the final ligand was obtained in 75% yield after semi-preparative HPLC-MS purification.

**Scheme 2:**
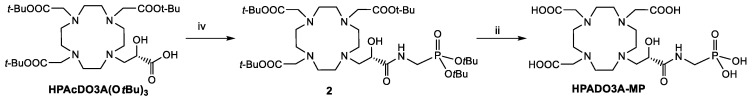
Synthesis of HPADO3A-MP. iv: *t-*Butyl-2-aminomethylphosphonate, HATU, DIPEA, 0 °C, 3 h; ii: TFA/DCM 1:1, RT, 18 h.

**Scheme 3:**
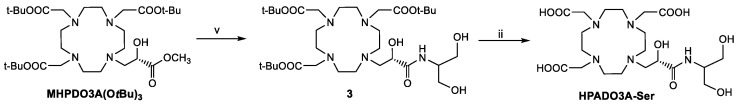
Synthesis of HPADO3A-Ser. v: 10 eq. serinol, MeOH, RT, 24 h; ii: TFA/DCM 1:1, 18 h.

Regarding the synthesis of the 2-hydroxypropylamide-based ligands (HPADO3As), the starting ligands for the synthesis were the DO3A(OtBu)_3_ bearing a 2-hydroxypropanoic acid pendant (HPAcDO3A(OtBu)_3_, Scheme 2) [22] in case of the derivatives with phosphonates peripheral groups and DO3A(OtBu)_3_ bearing the 2-hydroxypropanoate methyl ester (MHPDO3A(OtBu)_3_, Scheme 3) for the synthesis of the HPADO3A-Ser ligand. The intermediate **2** was obtained in 44% yield after semi-preparative HPLC-MS purification by coupling reaction of HPAcDO3A(OtBu)_3_ with *t-*Butyl-2-aminomethyl phosphonate [25] using the activator HATU (1-[Bis(dimethylamino)methylene]-1*H*-1,2,3-triazolo[4,5-*b*]pyridinium 3-oxid hexafluorophosphate) and DIPEA as base. This derivative **2** was then fully deprotected by using a 1:1 TFA/DCM mixture to obtain HPADO3A-MP. On the other hand, HPADO3A-Ser was obtained by aminolysis of the methyl ester of MHPDO3A(OtBu)_3_ with excess serinol, followed by TFA deprotection of the *tert*-butyl esters (Scheme 3) and semi-preparative HPLC-MS purification. ^1^H and ^13^C NMR and analytical HPLC-MS characterization of the final ligands are reported in the Supplementary Materials (Figures S9–S16).

**Scheme 4:**
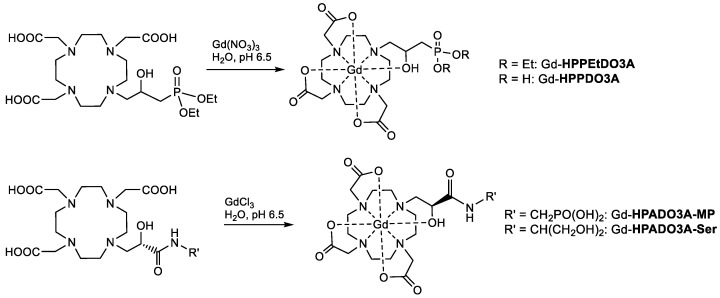
Synthesis of the Gd(III) complexes of the ligands reported in this work.

Finally, the Gd(III) complexes were synthesized by stirring an aqueous solution of the ligand and slight excess gadolinium chloride (or nitrate) at pH 6.5 and then by precipitating and filtering the unreacted Gd^3+^ at basic pH (Scheme 4). The complexes were characterized by ESI(+) mass spectrometry and the isotopic distribution was in excellent agreement with the theoretical data (Figures S1–S4).

### ^1^H Relaxometric Analysis of the Gd(III) Complexes

A detailed ^1^H NMR relaxometric study was carried out on all Gd(III) complexes. The relaxivity (*r*_1_) values of the five new Gd(III) complexes at 20 MHz and 298 K (Table 1) show a clear trend with higher values found for GdHPADO3A-MP, GdHPPDO3A, and GdHPADO3A-Ser, all having free polar hydrophilic peripheral groups with respect to the protected analogue GdHPPEtDO3A. The *r*_1_ values measured at 20 MHz, 298 K, and pH 7.4 are between 20 and 60% higher than those measured for the parent GdHPDO3A complex, i.e., the clinically approved GBCA Gadoteridol. This can be explained by the contribution of the phosphonate, amide, and hydroxyl groups to the relaxivity as a result of the catalysis of the proton exchange of the –OH proton and of the presence of second-sphere water molecules in interaction with the polar groups.

**Figure 3:**
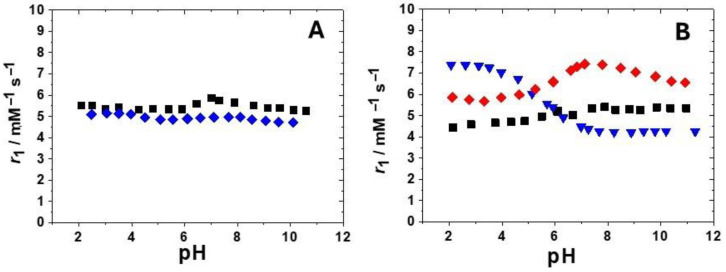
(**A**): comparison between the *r*_1_ values as a function of pH (20 MHz, 298 K) for GdHPPDO3A (black squares) and GdHPPEtDO3A (blue diamonds); (**B**): *r*_1_ values as a function of pH (20 MHz, 298 K) for GdHPADO3A-MP (red diamonds) and GdHPADO3A-Ser (black squares) compared to the variable pH profile reported for the secondary amide analogue GdBzHPADO3A [21] (blue triangles).

In order to evaluate the extent of these contributions, *r*_1_ was measured for all complexes as a function of pH. In fact, with GdHPADO3A [21], the presence of two amidic protons provided a strong contribution to the acid-catalyzed proton exchange to *r*_1_. In fact, a relaxivity value of 9.8 mM^−1^ s^−1^ at pH 4.2 and 298 K that decreases to 4.3 mM^−1^ s^−1^ at pH 7.4 was reported [21]. In the case of the ligand bearing the 2-hydroxypropylphosphonate pendant arm, we compared the *r*_1_ vs. pH profiles of the phosphonate-protected and deprotected derivatives with the aim of evaluating the effect of the free polar phosphonate group on *r*_1_ (Figure 3A). This effect seems not very pronounced across all pH values. In any case, all profiles, except that of GdHPPEtDO3A, show a small *r*_1_ increase at around neutral pH, indicating a small acid- or base-catalyzed proton exchange contribution to the relaxivity in addition to the second sphere effect. In Figure 3B, the *r*_1_ vs. pH profile of GdBzHPADO3A, the DO3A-hydroxypropylamide derivative with a secondary amide group responsible for the proton exchange of the OH group (Figure 1) [21], was also reported with the aim to compare it with the two new hydroxypropylamide-based Gd(III) complexes. The behavior seems completely different owing to a reduced or quenched acid-catalyzed proton exchange in the case of the serinol derivative and, thus, a more relevant base-catalyzed proton exchange and second-sphere contribution to the relaxivity. Interestingly, GdHPADO3A-MP shows higher relaxivity at 298 K, 20 MHz, and pH 7.4 (*r*_1_ = 7.4 mM^−1^ s^−1^) among all the reported complexes, probably due to the synergic contribution of the secondary amide and of the phosphonate group to the OH proton exchange and second sphere contributions. The *r*_1_ value of GdHPADO3A-MP is quite similar to those reported for GdAHPDO3A-P1 and P2 (Figure 1, 7.1 and 8.3 mM^−1^ s^−1^ in saline, at 20 MHz and 310 K, respectively), where the hydroxyl function is close to an amino (secondary and tertiary amine, respectively) and a phosphonate group [19]. In those cases, a strong contribution of the base-catalyzed proton exchange process was postulated thanks to an H-bond formed between the metal-coordinated OH and the deprotonated phosphonate group [19].

**Figure 4:**
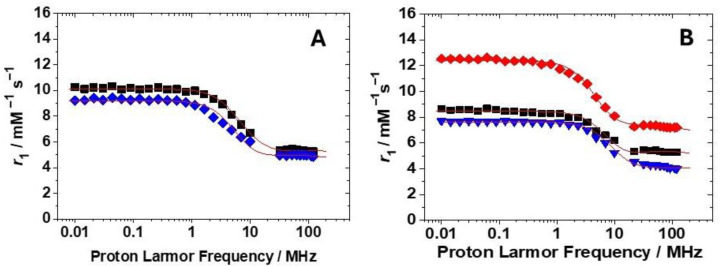
(**A**): ^1^H NMRD profiles at 298 K for GdHPPDO3A (black squares) and GdHPPEtDO3A (blue diamonds); (**B**): ^1^H NMRD profiles at 298 K for GdHPADO3A-MP (red diamonds), GdHPADO3A-Ser (black squares), and GdBzHPADO3A (blue triangles). The solid lines correspond to the fits of the data as described in the text.

The magnetic field dependence of *r*_1_ (Nuclear Magnetic Relaxation Dispersion (NMRD) profiles) was measured for the four new Gd-complexes at pH 7.4 at both 298 and 310 K in the proton Larmor frequency range 0.01–70 (or 120) MHz, corresponding to magnetic field strengths varying between 2.34 × 10^−4^ T and 3.0 T (Figure 4 and Figures S5–S8). The lower relaxivity values measured at 310 K across the entire range of proton Larmor frequencies studied suggest that *r*_1_ is limited by rotational motion rather than by the water exchange rate, indicating a fast exchange regime. With the aim to obtain important physicochemical parameters useful to understand the solution behaviour of these complexes, a least-squares fit of the data was carried out using the established theory of paramagnetic relaxation, described by the Solomon–Bloembergen–Morgan equations [26] for the inner sphere (IS) mechanism and the Freed equations [27] for the outer sphere (OS) mechanism. Given that the fitting process involves numerous parameters, some were fixed at known or reasonable values to facilitate the analysis. Thus, the hydration number *q* was fixed to 1; the distance of the closest approach, *a*, of the outer sphere water molecules to Gd^3+^ was set to 4.0 Å; the distance between the metal ion and the bound water protons, *r*, was fixed to 3.0 Å; and for the relative diffusion coefficient *D*, the standard values of 2.24 and 3.1 × 10^−5^ cm^2^ s^−1^ (298 and 310 K) were considered. The fit was performed using adjustable parameters *τ*_R_ and the electronic relaxation parameters Δ^2^ (the mean square transient ZFS energy) and its correlation time *τ*_V_. Since the complexes are in the fast exchange regime, the lifetime of the coordinated water molecule was fixed to that reported for similar GdHPADO3A complexes (*τ*_M_ = 24.2 ns) [21]. Except for GdHPPDO3AEt, where the phosphonate group is protected and the interaction with water molecules is weaker, the NMRD profiles of the other Gd(III) complexes were fitted also considering the contribution of second-sphere water molecules, i.e., water molecules H-bonded to the complex at a quite short distance from Gd^3+^ (ca. < 4 Å) and with a residence time sufficiently long to be affected by the rotation [28]. This contribution is expressed in terms of three additional parameters: the number *q*^SS^ of second-sphere water molecules, their mean distance from the metal ion, and their rotational correlation time, τ_R(SS)_. Two of these parameters were fixed: *q*^SS^ = 4, the same value reported for GdBzHPADO3A, and their distance from the metal ion: *r*^GdH(SS)^ = 3.5 Å, the same value considered in previous studies [21,29]. Such SS contribution to the relaxivity was also previously reported for the GdHPADO3A-derivatives [21] and for the dimeric Gd_2_(HPADO3A)_2_ complex [29], where the increase in *r*_1_ at pH 7.4 was explained by taking into account the contribution of 3 to 5 SS water molecules depending on the structure. 

For the complexes herein discussed, the rotational correlation times were found to be all in the same order of magnitude, between 80 and 90 ps, consistent with the increased molecular weight with respect to GdHPADO3A complexes. Furthermore, the electron relaxation parameters, *τ*_V_ and Δ^2^, agree with those obtained for other GdHPADO3A-like complexes. Finally, the rotational correlation times of the SS water molecules in interaction with the polar groups of the complexes (*τ*_RSS_ = 8–12 ps) are only slightly lower than the values reported for the GdHPADO3A analogs.

## 3. Materials and Methods

### 3.1. General

All chemicals, unless specified otherwise, were obtained from Sigma-Aldrich (Merck KGaA, Darmstadt, Germany) and used without further purification. Di-tert-Butyl-2-aminomethyl phosphonate was synthesized as reported in the literature [25]. The ^1^H and ^13^C NMR spectra were recorded on a Bruker Advance III 500 MHz (11.4 T) spectrometer equipped with a 5 mm PABBO probe and a BVT-3000 temperature control unit (Bruker, Billerica, MA, USA). Chemical shifts (δ) are referenced to TMS, with residual solvent proton resonances used for calibration. HPLC analyses and mass spectra were conducted using a Waters HPLC-MS system with a Waters 1525 binary pump. Preparative HPLC-MS was performed on a Waters C18 XTerra Prep column (5 μm, 19 × 50 mm) following the methods outlined in the Supplementary Materials. Electrospray ionization mass spectra (ESI-MS) were acquired using a Waters SQD 3100 Mass Detector, operating in positive or negative ion mode, with 1% *v*/*v* formic acid in methanol as the carrier solvent (Waters Corporation, Milford, MA, USA). 

### 3.2. Synthesis of 10-[3-Diethylphosphinyl-2-hydroxypropyl]-1,4,7,10-tetraazacyclododecane-1,4,7-triacetic Acid Tris-tert-butyl Ester (***1***)

A total of 263 mg (0.51 mmol) of DO3A(O-*t*Bu)_3_ and diethyl (2-oxiranylmethyl)phosphonate (700 mg, 3.6 mmol) was dissolved in *tert*-butanol and the mixture was stirred at reflux temperature overnight. After that, the solvent was evaporated under reduced pressure, and the crude was purified by HPLC-MS (method 1) to obtain 108.5 mg of compound **1** (30% yield). ^1^H NMR (500 MHz, CDCl_3_, 25 °C): δ (ppm) 1.29 (m, 6H, -OCH_2_-C*H_3_*), 1.41 (s, 27H, -C(C*H_3_*)_3_), 2.09 (m, 2H, -C*H_2_*-PO-), 2.8–4.08 (m, 28H, macrocycle, -C*H_2_*-COO*t*Bu, -OC*H_2_*-CH_3_), 4.45 (m, 1H, -CH_2_-C*H*(OH)-). ^13^C NMR (125 MHz, CDCl_3_, 25 °C): δ (ppm) 16.2 (-OCH_2_-*C*H_3_), 27.9 (-*C*(CH_3_)_3_), 31.6 (*J* = 138 Hz, -*C*H_2_-PO-), 47.6–54.2 (macrocycle), 55.0 (-*C*H_2_-CO-), 58.3 (-*C*H_2_-CH(OH)-), 62.5 (-O*C*H_2_-CH_3_), 83.1 (-*C*(CH_3_)_3_), 165.5 (-CH_2_-*C*OOC(CH_3_)_3_). ^31^P NMR (202 MHz, CDCl_3_, 25 °C): δ (ppm) 27.55. ESI-MS (*m*/*z*): found 709.90 [M + H]^+^ (calc for C_33_H_65_N_4_O_10_P: 708.87).

### 3.3. Synthesis of 10-[3-Diethylphosphinyl-2-hydroxypropyl]-1,4,7,10-tetraazacyclododecane-1,4,7-triacetic Acid (HPPEtDO3A)

A total of 38 mg of compound **1** (0.054 mmol) were dissolved in CH_2_Cl_2_ (1 mL), and 1 mL of trifluoroacetic acid was added dropwise. The solution was stirred at room temperature overnight, and then the solvent was removed from the rotary evaporator. The ligand HPPEtDO3A was obtained as the trifluoroacetate salt after precipitation with diethyl ether (^1^H NMR (500 MHz, D_2_O, 25 °C): δ (ppm) 1.27 (m, 6H, -OCH_2_-C*H_3_*), 2.20 (m, 2H, -C*H_2_*-PO-), 3.00–3.98 (m, 28H, macrocycle, -C*H_2_*-COOH), 4.11 (q, 4H, -OC*H_2_*-CH_3_), 4.39 (m, 1H, -CH_2_-C*H*(OH)-). ^13^C NMR (125 MHz, D_2_O, 25 °C): δ (ppm) = 15.6 (-OCH_2_-*C*H_3_), 30.7 (*J* = 138 Hz, -*C*H_2_-PO-), 47.2–54.4 (macrocycle), 58.3 (-*C*H_2_-COOH), 61.6 (-*C*H_2_-CH(OH)-), 63.7 (-O*C*H_2_-CH_3_)), 173.6 (-CH_2_-*C*OOH). ^31^P NMR (202 MHz, D_2_O, 25 °C): δ (ppm) = 31.06. HPLC/MS: A (TFA 0.1% in H_2_O); B (ACN); flow 1 mL/min; 0–5 min: 1% B, 5–15 min: from 30 a 100% B, 15–19 min 100% B, 19–20 min from 100 a 1% B; t_r_ = 11.99 min. ESI-MS (*m*/*z*): found 541.47 [M + H]^+^ (calc for C_21_H_41_N_4_O_10_P: 540.55).

### 3.4. Synthesis of 10-[3-Phosphinyl-2-hydroxypropyl]-1,4,7,10-tetraazacyclododecane-1,4,7-triacetic Acid (HPPDO3A)

Bromo trimethylsilane (116 mg, 100 μL), diluted in CH_2_Cl_2_ (2 mL), was added dropwise to an ice-bath-cooled solution of compound **1** (70 mg, 0.1 mmol) in CH_2_Cl_2_ (2 mL). After 15 min, the ice bath was removed, and the mixture was stirred at room temperature overnight. The dichloromethane was then evaporated, and the crude ligand was obtained after precipitation using diethyl ether. The final ligand HPPEtDO3A was obtained as a trifluoroacetate salt (50 mg, quantitative yield). ^1^H NMR (500 MHz, D_2_O, 25 °C): δ (ppm) 1.93 (m, 2H, -C*H_2_*-PO-), 3.09–3.89 (m, 28H, macrocycle, -C*H_2_*-COOH), 4.36 (m, 1H, -CH_2_-C*H*(OH)-). ^13^C NMR (125 MHz, D_2_O, 25 °C): δ (ppm) 34.3 (*J* = 138 Hz, -*C*H_2_-PO-), 47.0–51.5 (macrocycle), 58.9 (-*C*H_2_-COOH), 62.30 (-*C*H_2_-CH(OH)-), 174.1 (-CH_2_-*C*OOH). ^31^P NMR (202 MHz, D_2_O, 25 °C): δ (ppm) 20.7. HPLC/MS: A (TFA 0.1% in H_2_O); B (ACN); flow 1 mL/min; 0–5 min: 1% B, 5–15 min: from 30 a 100% B, 15–19 min 100% B, 19–20 min from 100 a 1% B; t_r_ = 2.71 min. ESI-MS (*m*/*z*): found 485.37 [M + H]^+^ (calc for C_17_H_33_N_4_O_10_P: 484.44).

### 3.5. Synthesis of 10-[Di-Tert-butylphosphonomethyl-2-hydroxy-3-oxypropyl]-1,4,7,10-teraazacyclododecane-1,4,7-triacetic Acid Tris-tert-butyl Ester (***2***)

The *t*-Bu-protected ligand HPAcDO3A(OtBu)_3_ (56 mg, 0.13 mmol) dissolved in DMF (5 mL) was reacted with 1.2 eq of di-*tert*-butyl aminomethylphosphonate (34.7 mg, 0.156 mmol) in the presence of 1.2 eq di HATU (59 mg, 0,156 mmol) and 1.5 eq di DIPEA (34 μL, 0.2 mmol). The reaction was carried out at 0 °C for 3 h, and then the solvent was evaporated. The crude product was purified by HPLC-MS (method 2, RT = 16.7 min) to obtain 43 mg (0.057 mmol, 44% yield) of the product. ^1^H NMR (500 MHz, CDCl_3_ 25 °C):δ (ppm) 4.67 (m, -N-CH_2_-C*H*(OH)-CO-, 1H), 3.98 (m, 2H, -N-C*H_2_*-CH(OH)-CO-), 3.68 (m, 6H, N-C*H_2_*-COOH, 6H), 3.60–3.14 (m, 16H, macrocycle), 2.74 (d, 2H, *J* = 9.5 Hz), -NH-C*H_2_*-P(OH)_2_), 1.43 (s, 45H, -N-CH_2_-COOC(C*H_3_*)_3_). ^13^C NMR (125MHz, CDCl_3_, 25 °C): δ (ppm) 168.3 (N-CH_2_-CH(OH)-*CO*-), 169.4 (-N-CH_2_-*C*OOC(CH_3_)_3_),82.3 (-N-CH_2_-COO*C*(CH_3_)_3_), 65.6 (-N-CH_2_-*C*H(OH)-CO-), 54.8 (N-*C*H_2_-CH(OH)-CO-), 53.9 (N-*C*H_2_-COOC(CH_3_)_3_), 50.4–47.8 (macrocycle), 36.9 (*J* = 147 Hz, -NH-*C*H_2_-P(OH)_2_), 28.2 (-N-CH_2_-COOC(*C*H_3_)_3_). ESI-MS (*m*/*z*): found 809.01 [M + H]^+^ (calc for C_38_H_74_N_5_O_11_P: 807.99).

### 3.6. Synthesis of 10-[Phosphonomethyl-2-hydroxy-3-oxypropyl]-1,4,7,10-tetraazacyclododecane-1,4,7-triacetic Acid (HPADO3A-MP)

A total of 50 mg of the protected ligand (0.062 mmol) were dissolved in a 1:1 mixture of CH_2_Cl_2_ and TFA, and the solution was stirred at room temperature overnight. Then, the solution was evaporated, and the crude product was purified by HPLC-MS (method 3, RT = 1.45 min) to obtain 41.8 mg of the final ligand (21 mg, 0.040 mmol, 64.5% yield). ^1^H NMR (500 MHz, D_2_O, 25 °C): δ (ppm) = 4.66 (m, -N-CH_2_-C*H*(OH)-CO-, 1H), 4.01 (m, 2H, -N-C*H_2_*-CH(OH)-CO-), 3.71 (m, 6H, N-C*H_2_*-COOH, 6H), 3.55–3.24 (m, 16H, macrocycle), 2.72 (s, 2H, *J* = 9.5 Hz), (-NH-C*H_2_*-P(OH)_2_). ^13^C NMR (125MHz, D_2_O, 25 °C): δ (ppm) = 172.6 (N-CH_2_-CH(OH)-*C*O-), 172.4 (-N-CH_2_-*C*O-OH), 66.9 (-N-CH_2_-*C*H(OH)-CO-), 55.3 (N-*C*H_2_-CH(OH)-CO-), 54.6 (N-*C*H_2_-COOH), 53.5–48.8 (macrocycle), 36.9 (*J* = 147 Hz, -NH-*C*H_2_-P(OH)_2_). ESI-MS (*m*/*z*): found 528.26 [M + H]^+^ (calc for C_18_H_34_N_5_O_11_P: 527.20).

### 3.7. Synthesis of 10-[Bis-(1,3-dihydroxypropan-2-yl)-2-hydroxy-3-oxypropyl]-1,4,7,10-tetraazacyclododecane-1,4,7-triacetic Acid Tris-tert-butyl Ester (***3***)

HPADO3A-methyl ester (50 mg, 0.08 mmol) and 10 eq of 2-amino-1,3-propandiol (serinol, 72.84 mg, 0.8 mmol) were dissolved in 5 mL of MeOH and stirred at room temperature for 24 h. The presence of the product was verified by mass spectrometry. The product was purified by semi-preparative HPLC-MS (Method 4, RT = 11.55 min) to obtain 30 mg (0.045 mmol) of the product with 56% yield. ^1^H NMR (500 MHz, CD_3_CN, 25 °C): δ (ppm) = 4.76 (m, -N-CH_2_-C*H*(OH)-, 1H), 4.12 (m, 1H, -CO-NH-C*H*(CH_2_OH)_2_), 4.01 (m, 2H, -N-C*H_2_*-CH(OH)-), 3.75 (m, 4H, -CO-NH-CH(C*H_2_*OH)_2_), 3.67 (m, 6H, N-C*H_2_*-COO*t*Bu, 6H), 3.50–3.11 (m, 16H, macrocycle), 1.43 (s, 27H, -C(C*H_3_*)_3_). ^13^C NMR (125MHz, CD_3_CN, 25 °C): δ (ppm) = 174.3 (N-CH_2_-CH(OH)-*C*O-), 170.7 (N-CH_2_-*C*O-OC(C*H_3_*)_3_), 83.2 (*C*(CH_3_)_3_), 66.6 (N-CH_2_-*C*H(OH)), 62.5 (-CO-NH-*C*H(CH_2_OH)_2_), 58.6 (-CO-NH-CH(*C*H_2_OH)_2_), 54.9 (N-*C*H_2_-CH(OH)), 54.2 (N-*C*H_2_-COO*t*Bu), 53.2-47.7 (macrocycle), 28.25 (-C(*C*H_3_)_3_). ESI-MS (*m*/*z*): found 676.56 [M + H]^+^ (calc for C_32_H_61_N_5_O_10_: 675.44).

### 3.8. Synthesis of 10-[Bis-(1,3-dihydroxypropan-2-yl)-2-hydroxy-3-oxypropyl]-1,4,7,10-tetraazacyclododecane-1,4,7-triacetic Acid (HPADO3A-Ser)

HPADO3A(O*t*Bu)_3_-Ser (30 mg, 0.045 mmol) was dissolved in a 1:1 mixture of DCM and TFA and stirred at RT overnight. After removal of the solvents at reduced pressure, the residue was taken up with a solution of HCl (1 M) and brought to dryness in order to exchange the trifluoroacetate salt with HCl (quantitative yield). ^1^H NMR (500 MHz, D_2_O, 25 °C): δ (ppm) = 4.69 (m, -N-CH_2_-C*H*(OH)-, 1H), 4.09 (m, 1H, -CO-NH-C*H*(CH_2_OH)_2_), 3.97 (m, 2H, -N-C*H_2_*-CH(OH)-), 3.68 (m, 4H, -CO-NH-CH(C*H_2_*OH)_2_), 3.55 (m, N-C*H_2_*-COOH, 6H), 3.47–2.97 (m, 16H, macrocycle). ^13^C NMR (125MHz, D_2_O, 25 °C): δ (ppm) = 174.3 (CH(OH)-*C*O), 173.7 (N-CH_2_-*C*OOH), 66.6 (N-CH_2_-*C*H(OH)-CO), 62.5 (-CO-NH-*C*H(CH_2_OH)_2_), 58.6 (-CO-NH-CH(*C*H_2_OH)_2_), 54.9 (N-*C*H_2_-CH(OH)-CO-), 54.2 (N-*C*H_2_-COOH), 53.2–47.7 (macrocycle). ESI-MS (*m*/*z*): found 508.26 [M + H]^+^ (calc for C_20_H_37_N_5_O_10_: 507.25).

### 3.9. General Procedure for the Preparation of Gd(III) Complexes

The ligands (20 mg) were dissolved in H_2_O (1 mL), and GdCl_3_ or Gd(NO_3_)_3_ (1.05 eq.) was added, maintaining the pH around 6.5–7, and the solution was stirred overnight at room temperature. The pH was then raised to 9.5 to allow precipitation of excess gadolinium as Gd(OH)_3,_ and after 2 h, the solution was filtered and lyophilized after acidification to pH 7 to obtain the final complex.

### 3.10. Relaxation Measurements

^1^H nuclear magnetic relaxation dispersion (NMRD) profiles were collected over a continuum of proton Larmor Frequencies magnetic (0.01–120 MHz). A Fast-Field Cycling (FFC) Stelar SmarTracer Relaxometer (Stelar srl, Mede (PV), Italy) equipped with a silver magnet was used to acquire the NMRD profiles at lower frequencies (0.01–10 MHz). A high-field relaxometer (Stelar) equipped with an HTS-110 3T Metrology cryogen-free superconducting magnet was used to acquire the data at higher frequencies (20–120 MHz). The data were collected using the standard inversion recovery sequence (20 experiments and 2 scans) with a typical 90° pulse width of 3.5 μs, and the reproducibility of the data was within ±0.5%. The temperature was controlled with a Stelar VTC-91 heater airflow (Stelar) equipped with a copper-constantan thermocouple (uncertainty of ±0.1 K).

## 4. Conclusions

An investigation into the influence of different peripheral functional groups (amides, phosphonates, diols) on the relaxivity of GdHPDO3A-like complexes was reported in this work. Importantly, GdHPDO3A-like complexes share the same macrocyclic coordination cage, guaranteeing high thermodynamic stability and kinetic inertness of the Gd(III) complexes, as demonstrated by previous studies [13,14,15,16,17,18]. The synthesis of new Gd(III) complexes with a DO3A ligand bearing a 2-hydroxypropylphosphonate pendant (with the phosphonate group protected and deprotected) or *N*-methylphosphonate or *N*-dihydroxypropyl-2-hydroxypropanamide arms was described. The relaxometric analysis of the Gd(III) complexes, including ^1^H relaxivity as a function of pH and NMRD profiles, showed that the presence of the polar groups adjacent to the coordinated hydroxyl functionality allowed an increase in the relaxivity at physiological pH. This *r*_1_ increase was ascribed to the presence of second-sphere water molecules in interaction with the polar groups close to the paramagnetic metal center and to an OH-proton exchange process that is accelerated by these polar groups. In conclusion, this work further confirms that peripheral protic functional groups adjacent to the donor atoms coordinating the Gd^3+^ ion are involved in the relaxivity enhancement thanks to a network of H-bonds formed between these functional groups and second-sphere water molecules.

## Figures and Tables

**Table 1:** Selected best-fit parameters obtained from the analysis of the ^1^H NMRD profiles (298 K, pH 7.4) data for the Gd(III) complexes of HPPDO3A, HPPEtDO3A, HPADO3A-MP, and HPADO3A-Ser ligand. The data are compared with those of GdBzHPADO3A ^a^.

Parameter	HPPDO3A	HPPEtDO3A	HPADO3A-MP	HPADO3A-Ser	BzHPADO3A ^b^
*r*_1_ (mM^−1^ s^−1^) 20 MHz	5.7 ± 0.1	5.0 ± 0.1	7.4 ± 0.1	5.4 ± 0.1	4.5
*τ*_M_ (ns)	24.2 ^c^	24.2 ^c^	24.2 ^c^	24.2 ^c^	24.2
*τ*_R_ (ps)	80 ± 2	84 ± 2	86 ± 3	86 ± 2	70.8
Δ^2^ (10^19^ s^−2^)	14.0 ± 0.2	6.2 ± 0.1	6.6 ± 0.2	14.1 ± 0.2	10.2
*τ*_V_ (ps)	7.5 ± 0.3	13.7 ± 0.2	13.2 ± 0.3	10.0 ± 0.3	13.1
*q*_SS_	4 ^c^	--	4 ^c^	4 ^c^	4
*τ*_R(SS)_ (ps)	10.0 ± 0.2	--	12.1 ± 0.1	8.3 ± 0.2	24

^a^ Ref. [21]; ^b^ The parameters fixed in the fitting procedure are *q* = 1, *r*_GdO_ = 2.5 Å, r_GdH_ = 3.0 Å, a_GdH_ = 4.0 Å, ^298^D_GdH_ = 2.25 × 10^5^ cm^2^ s^−1^, and r_GdH(SS)_ = 3.5 Å. ^c^ Fixed to the value of the secondary amide analog GdBzHPADO3A.

## Data Availability

The data are available on request from the corresponding authors.

## Supplementary Materials

The following supporting information can be downloaded at: https://www.mdpi.com/article/10.3390/molecules29194663/s1, ^1^H NMRD profiles at 298 and 310 K of all Gd(III) complexes, HPLC-MS methods, equations used for fitting of NMRD profiles, HPLC-MS and NMR characterization of the ligands and ESI-MS spectra of the Gd(III) complexes. Refs. [30,31,32] are cited in the Supplementary Materials.
