# Supplementary material for: Relaxivity Modulation of Gd-HPDO3A-like Complexes by Introducing Polar and Protic Peripheral Groups
Source: Molecules. 2024 Sep 30;29(19):4663. doi: 10.3390/molecules29194663 (PMC11478047; doi:10.3390/molecules29194663)
Supplement: Supplementary file 1 [file molecules-29-04663-s001.zip › molecules-3229262-supplementary.pdf]

1) ESI mass spectra of the Gd(III) complexes

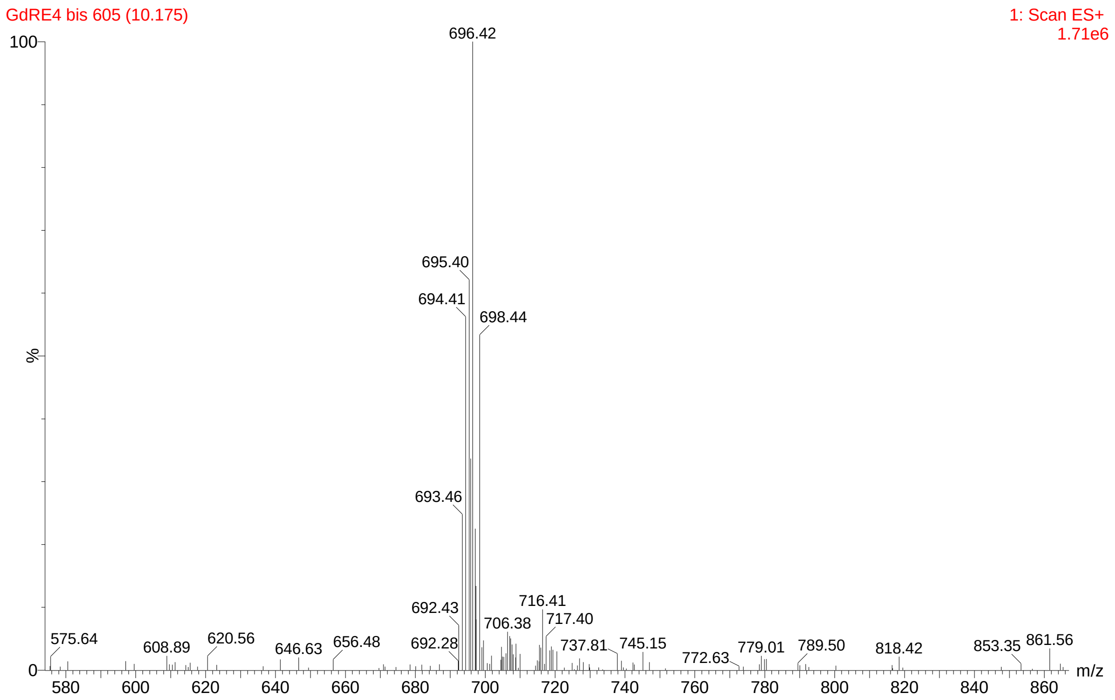

Figure S1. ESI(+) mass spectrum of GdHPPEtDO3A (calc. for $C_{21}H_{39}GdN_4O_{10}P$: 695.79).

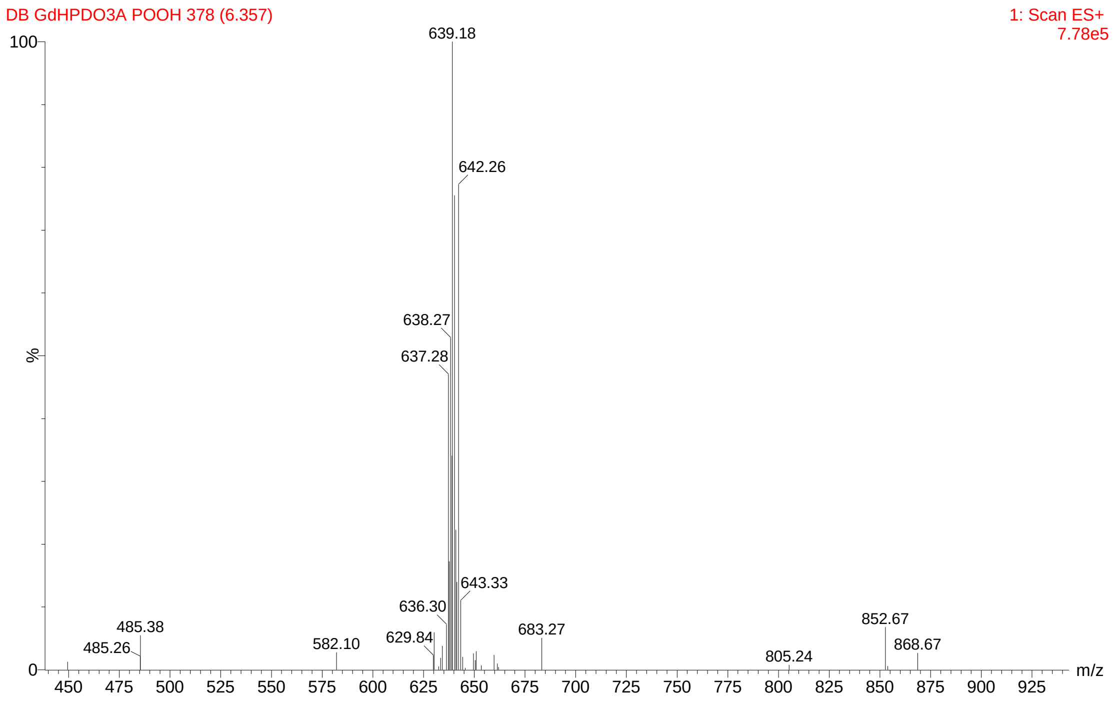

Figure S2. ESI(+) mass spectrum of GdHPPDO3A (calc. for $C_{17}H_{31}GdN_4O_{10}P$: 639.68).

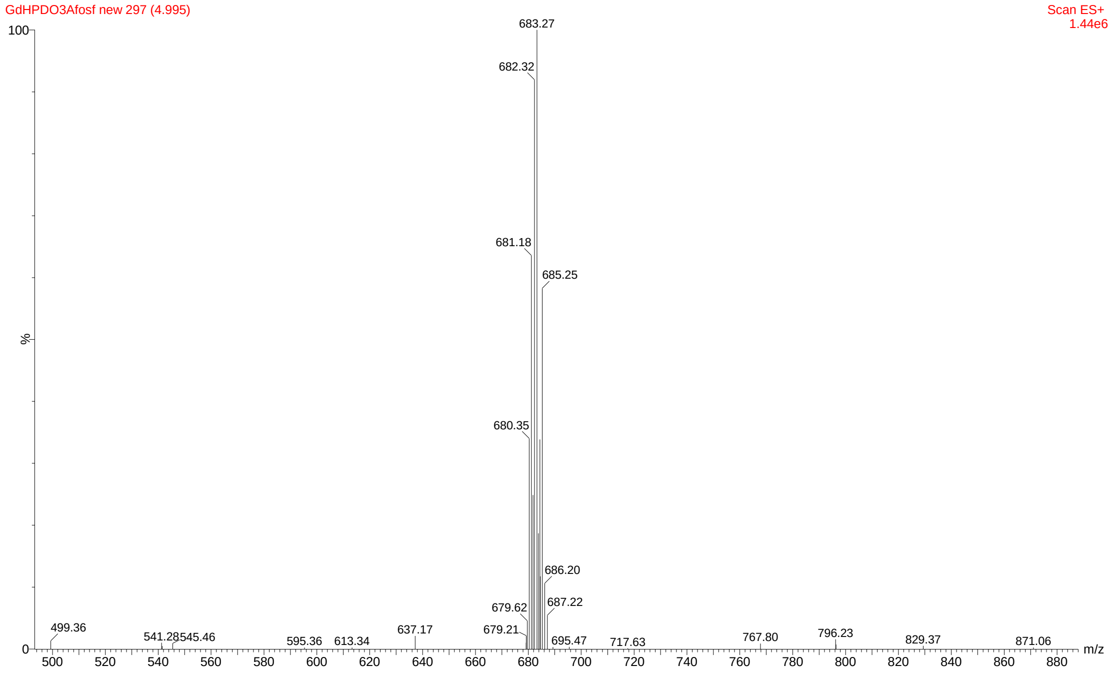

Figure S3. ESI(+) mass spectrum of GdHPADO3A-MP (calc. for $C_{18}H_{32}GdN_5O_{11}P$: 682.70).

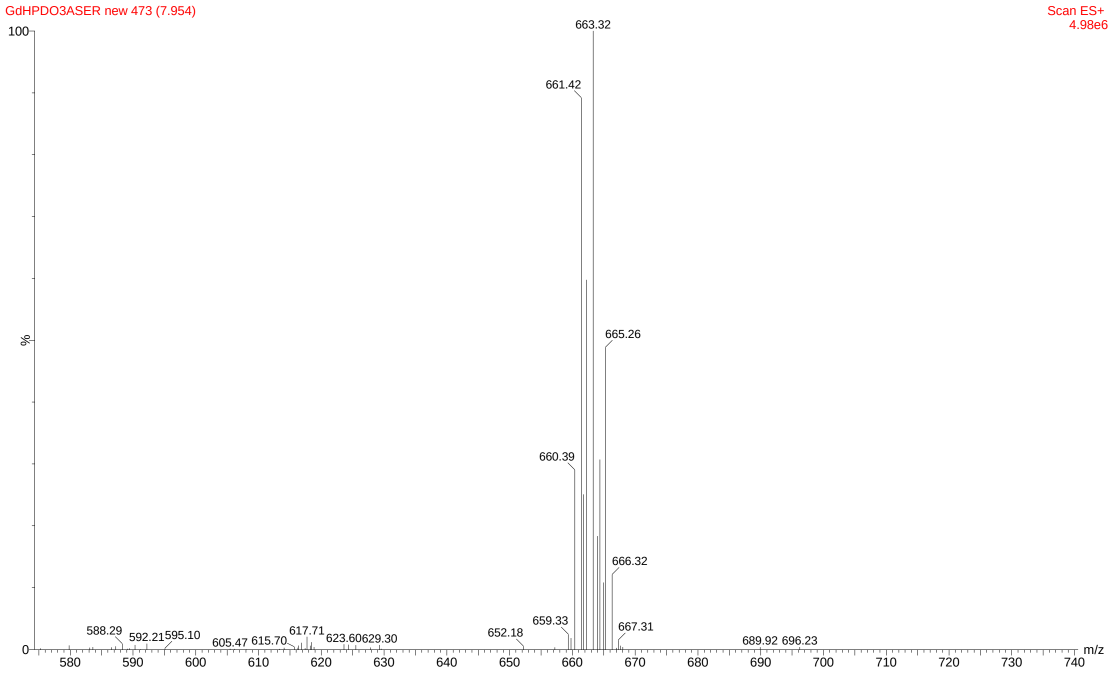

Figure S4. ESI(+) mass spectrum of GdHPADO3A-Ser (calc. for $C_{20}H_{35}GdN_5O_{10}$: 662.78).

2) HPLC-MS methods

Method 1

Time (min)	Flow (ml/min)	% A	% B
0.00	20.00	70.0	30.0
3.00	20.00	70.0	30.0
15.00	20.00	28.0	72.0
15.50	20.00	0.0	100.0
16.50	20.00	0.0	100.0
17.00	20.00	70.0	30.0
19.00	20.00	70.0	30.0

Method 2

Time (min)	Flow (mL/min)	% A	% B
0.00	20.00	70.0	30.0
2.00	20.00	70.0	30.0
27.00	20.00	0.0	100.0
28.00	20.00	0.0	100.0
29.00	20.00	70.0	30.0
30.00	20.00	70.0	30.0

Method 3

Time (min)	Flow (mL/min)	% A	% B
0.00	20.00	95.0	5.0
2.50	20.00	95.0	5.0
3.00	20.00	0.0	100.0
4.00	20.00	0.0	100.0
5.00	20.00	95.0	5.0
6.00	20.00	95.0	5.0

Method 4

Time (min)	Flow (mL/min)	% A	% B
0.00	20.00	70.0	30.0
3.00	20.00	70.0	30.0
23.00	20.00	0.0	100.0
24.00	20.00	0.0	100.0
25.00	20.00	70.0	30.0

Method 5

Time (min)	Flow (mL/min)	% A	% B
0.00	1.00	99.0	1.0
2.00	1.00	99.0	1.0
15.00	1.00	0.0	100.0
19.00	1.00	0.0	100.0
20.00	1.00	99.0	1.0

3) ^{1}H NMRD equations

The measured longitudinal proton relaxation rate, R_1^{obs} is the sum of a paramagnetic and a diamagnetic contribution as expressed in Eq. (7), where r_{1p} is the proton relaxivity:

$$R_1^{obs} = R_1^d + R_1^p = R_1^d + r_{1p}[Gd(III)] \qquad (7)$$

The relaxivity can be divided into an inner and an outer sphere term as follows:

$$r_1 = r_{1is} + r_{1os} \qquad (8)$$

The inner sphere term is given in Eq. (9), where q is the number of inner sphere water molecules.[i]

$$r_{1is} = \frac{1}{1000} \times \frac{q}{55.55} \times \frac{1}{T_{1m}^H + \tau_m} \qquad (9)$$

The longitudinal relaxation rate of inner sphere protons, $1/T_{1m}^{H}$ is expressed by Eq. (10):

$$\frac{1}{T_{1m}^H} = \frac{2}{15}\left(\frac{\mu_0}{4\pi}\right)^2 \frac{\gamma_I^2 g^2 \mu_B^2}{r_{MnH}^6} S(S+1)\left[\frac{3\tau_{d1}}{1+\omega_I^2\tau_{d1}^2} + \frac{7\tau_{d2}}{1+\omega_S^2\tau_{d2}^2}\right] \qquad (10)$$

where r_{MnH} is the effective distance between the electron charge and the ^{1}H nucleus, ω_I is the proton resonance frequency and ω_S is the Larmor frequency of the Gd(III) electron spin.

$$\frac{1}{\tau_{di}} = \frac{1}{\tau_m} + \frac{1}{\tau_R} + \frac{1}{T_{ie}} \qquad i = 1, 2 \qquad (11)$$

The longitudinal and transverse electronic relaxation rates, $1/T_{1e}$ and $1/T_{2e}$ are expressed by Eqs. (12)-(14),[ii] where τ_V is the electronic correlation time for the modulation of the zero-

field-splitting interaction, E_V the corresponding activation energy and Δ^2 is the mean square zero-field-splitting energy. We assumed a simple exponential dependence of τ_V versus $1/T$ as written in Eq. (14).

$$\frac{1}{T_{1e}} = \frac{1}{25}\Delta^2\tau_V\{4S(S+1)-3\}\left(\frac{1}{1+\omega_S^2\tau_V^2}+\frac{4}{1+4\omega_S^2\tau_V^2}\right) \qquad (12)$$

$$\frac{1}{T_{2e}} = \left(\left(0.02\times\left(4S^2+4S-3\right)\times\tau_V\times\Delta^2\times\left(\left(\frac{5}{1+\omega_S{}^2\tau_V{}^2}\right)\right)\right)+\left(\frac{2}{1+4\omega_S{}^2\tau_V{}^2}\right)+3\right) \qquad (13)$$

$$\tau_v = \tau_v^{298}\exp\left\{\frac{E_v}{R}\left(\frac{1}{T}-\frac{1}{298.15}\right)\right\} \qquad (14)$$

The outer-sphere contribution can be described by Eq. (15) where N_A is the Avogadro constant, and J_{os} is its associated spectral density function.[iii,iv]

$$r_{1os} = \frac{32N_A\pi}{405}\left(\frac{\mu_0}{4\pi}\right)^2\frac{\hbar^2\gamma_S^2\gamma_I^2}{a_{MnH}D_{MnH}}S(S+1)[3J_{os}(\omega_I;T_{1e})+7J_{os}(\omega_I;T_{2e})] \qquad (15)$$

$$J^{OS}\left(\omega,T_{je}\right)=\mathrm{Re}\left[\frac{1+\frac{1}{4}\left(i\omega\tau_{GdH}+\frac{\tau_{GdH}}{T_{je}}\right)^{1/2}}{1+\left(i\omega\tau_{GdH}+\frac{\tau_{GdH}}{T_{je}}\right)^{1/2}+\frac{4}{9}\left(i\omega\tau_{GdH}+\frac{\tau_{GdH}}{T_{je}}\right)+\frac{1}{9}\left(i\omega\tau_{GdH}+\frac{\tau_{GdH}}{T_{je}}\right)^{3/2}}\right] \qquad (16)$$

where $j=1,2$, $\tau_{GdH} = \frac{a_{GdH}{}^2}{D_{GdH}}$.

The diffusion coefficient for the diffusion of a water proton away from a Gd(III) complex, D_{GdH}, is assumed to obey an exponential law versus the inverse of the temperature, with an activation energy E_{GdH}, as given in Eq. (17). $D_{GdH}{}^{298}$ is the diffusion coefficient at 298.15 K.

$$D_{GdH} = D_{GdH}^{298}\exp\left\{\frac{E_{GdH}}{R}\left(\frac{1}{298.15}-\frac{1}{T}\right)\right\}$$

4) NMRD profiles

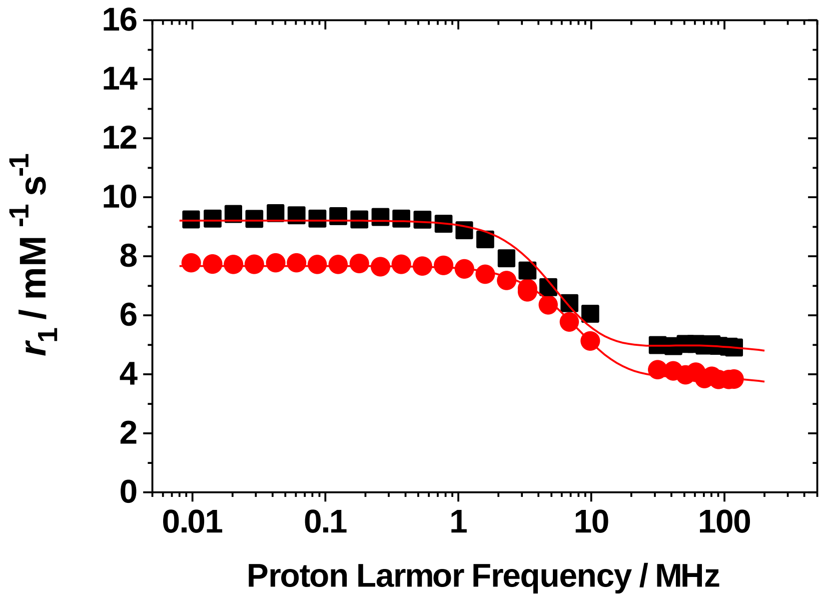

Figure S5. NMRD profiles at 298 (black squares) and 310 K (red circles) of GdHPPEtDO3A. The solid lines correspond to the fits of the data as described in the main text.

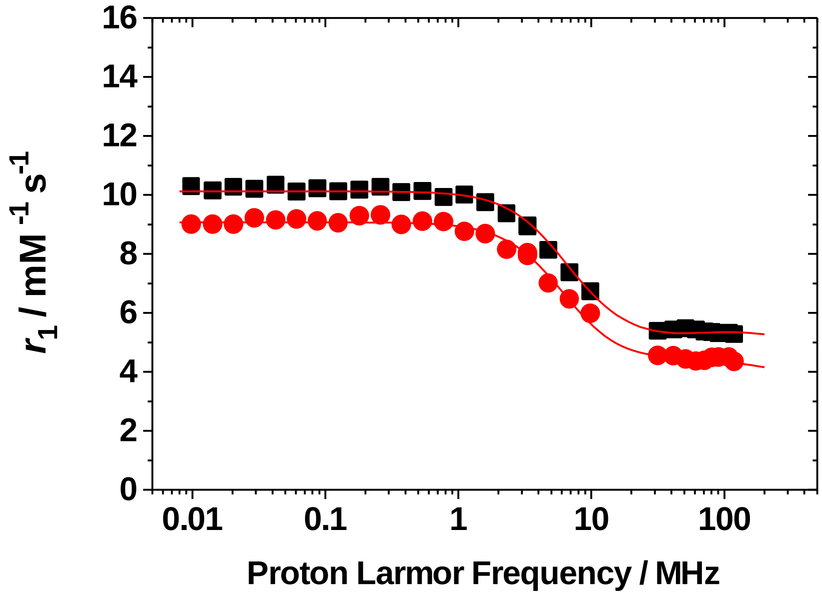

Figure S6. NMRD profiles at 298 (black squares) and 310 K (red circles) of GdHPPDO3A. The solid lines correspond to the fits of the data as described in the main text.

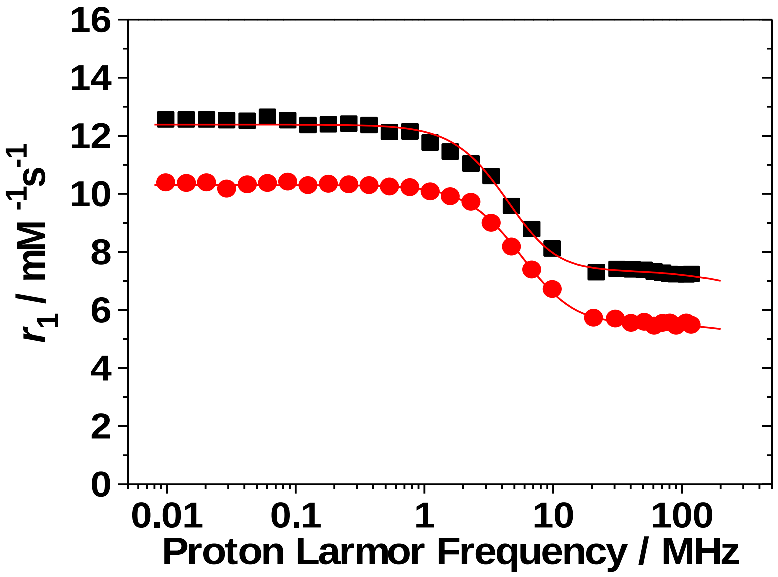

Figure S7. NMRD profiles at 298 (black squares) and 310 K (red circles) of GdHPADO3A-MP. The solid lines correspond to the fits of the data as described in the main text.

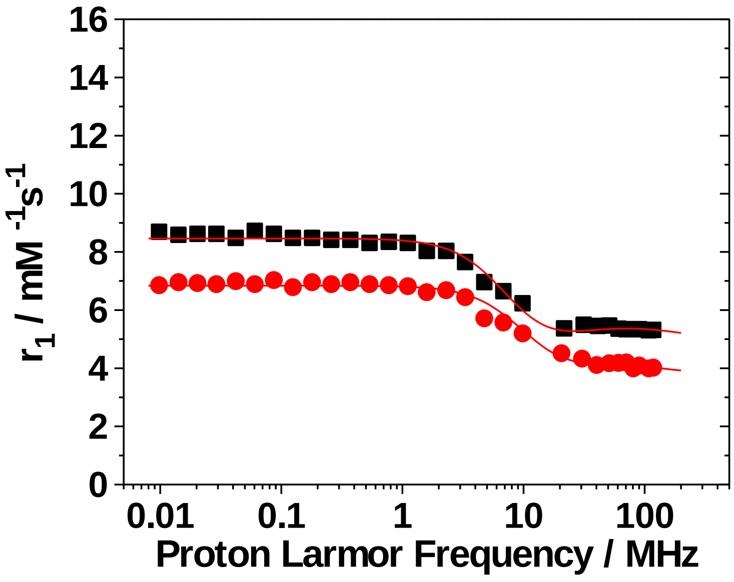

Figure S8. NMRD profiles at 298 (black squares) and 310 K (red circles) of GdHPADO3A-Ser. The solid lines correspond to the fits of the data as described in the main text.

5) HPLC-MS of the ligands:

HPPEtDO3A

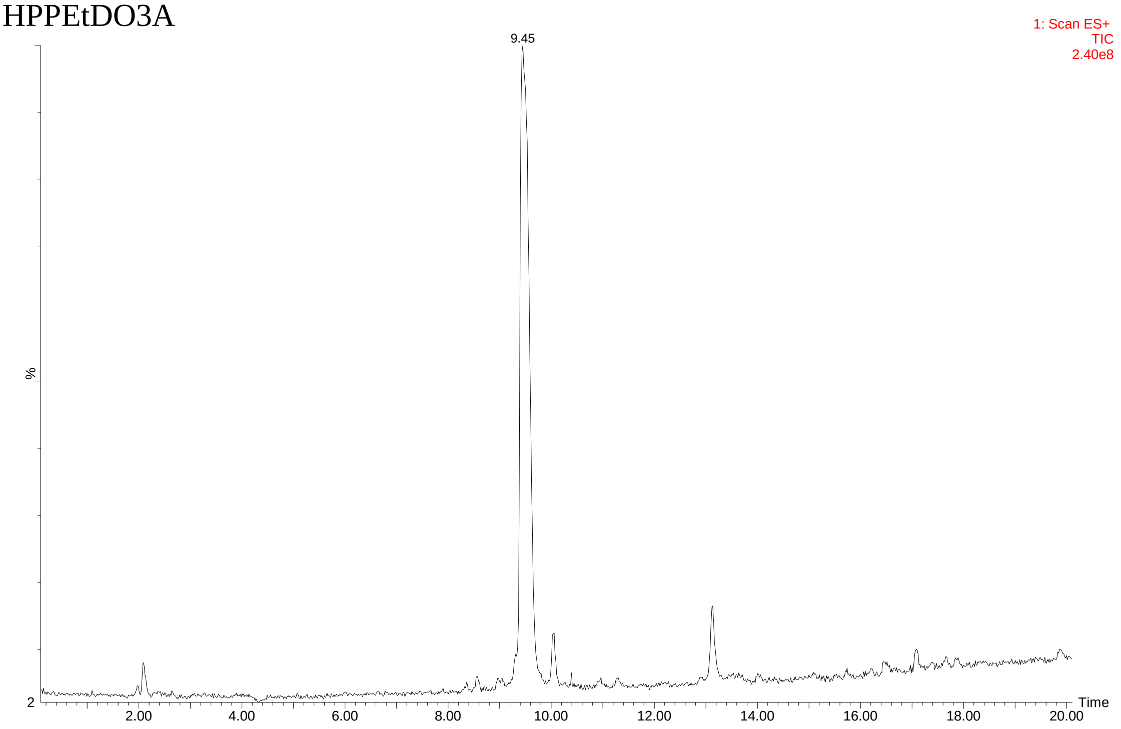

Figure S9. HPLC-MS of the ligand HPPEtDO3A (method 5, rt = 9.45)

HPPDO3A

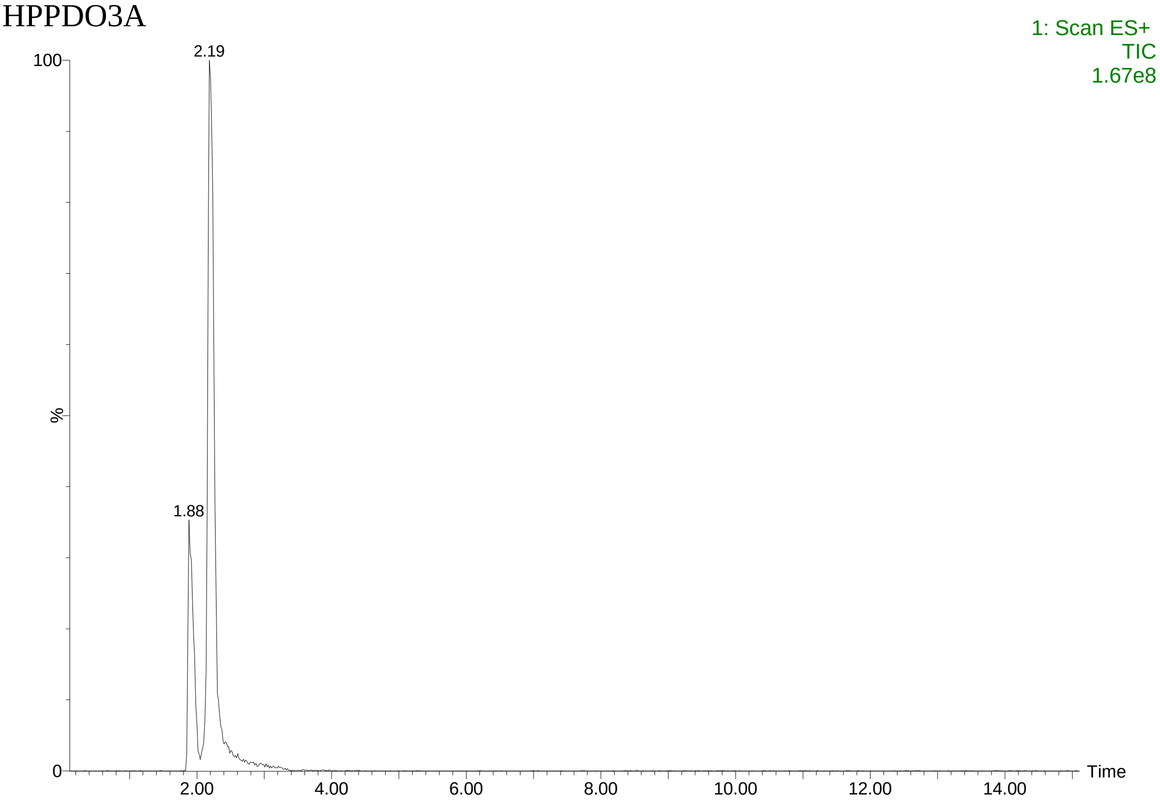

Figure S10. HPLC-MS of the ligand HPPDO3A (method 5, rt = 2.2)

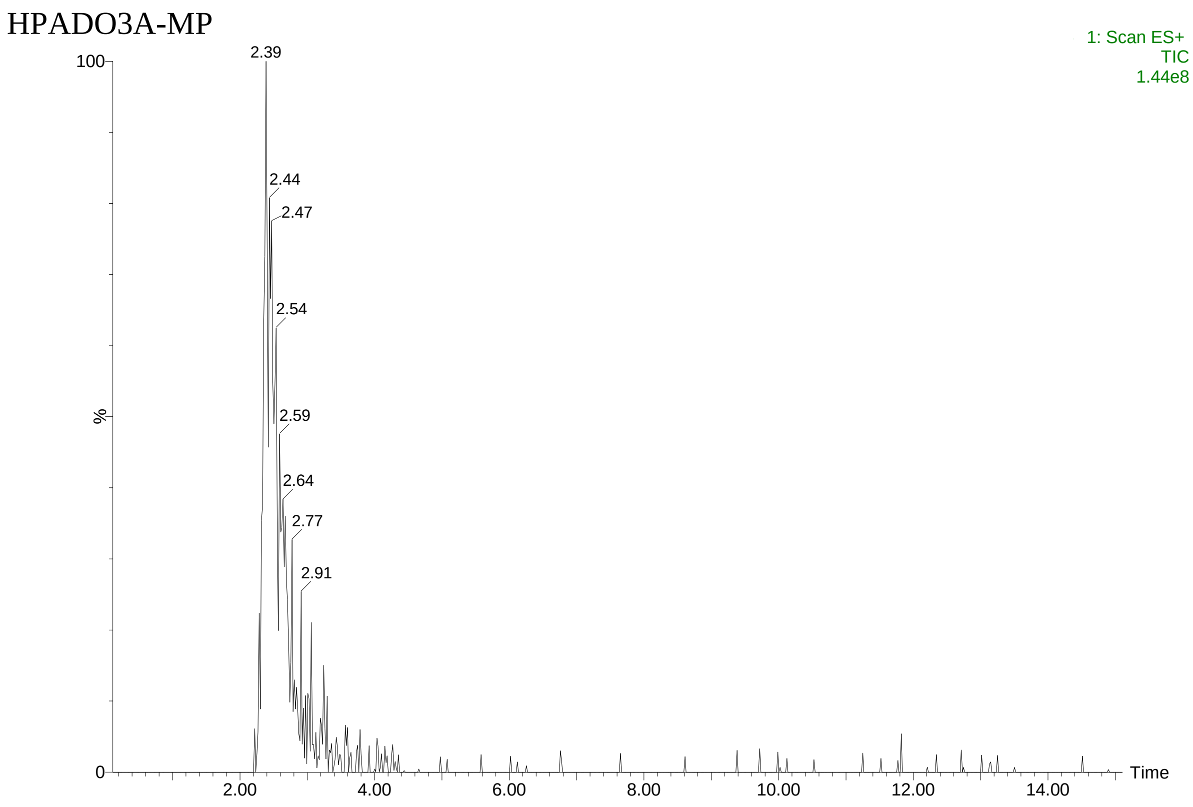

Figure S11. HPLC-MS of the ligand HPADO3A-MP (method 5, rt = 2.4)

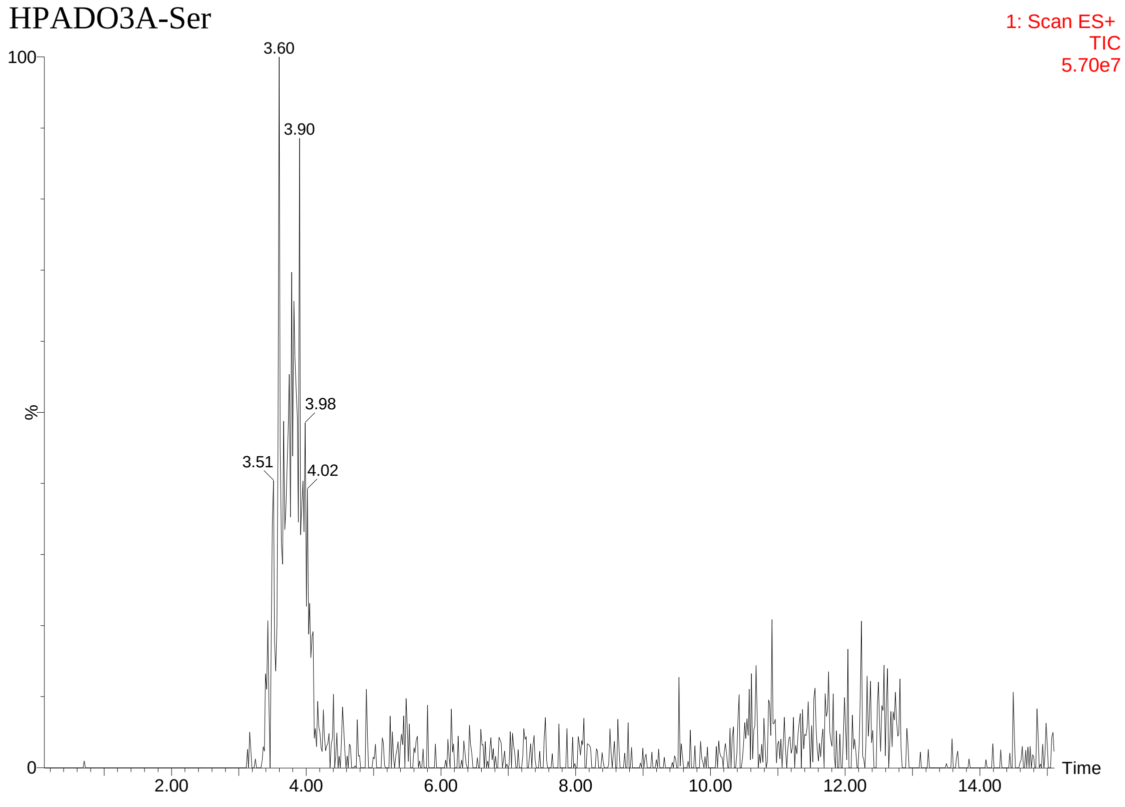

Figure S12. HPLC-MS of the ligand HPADO3A-Ser (method 5, rt = 3.7)

6) NMR spectra of the ligands

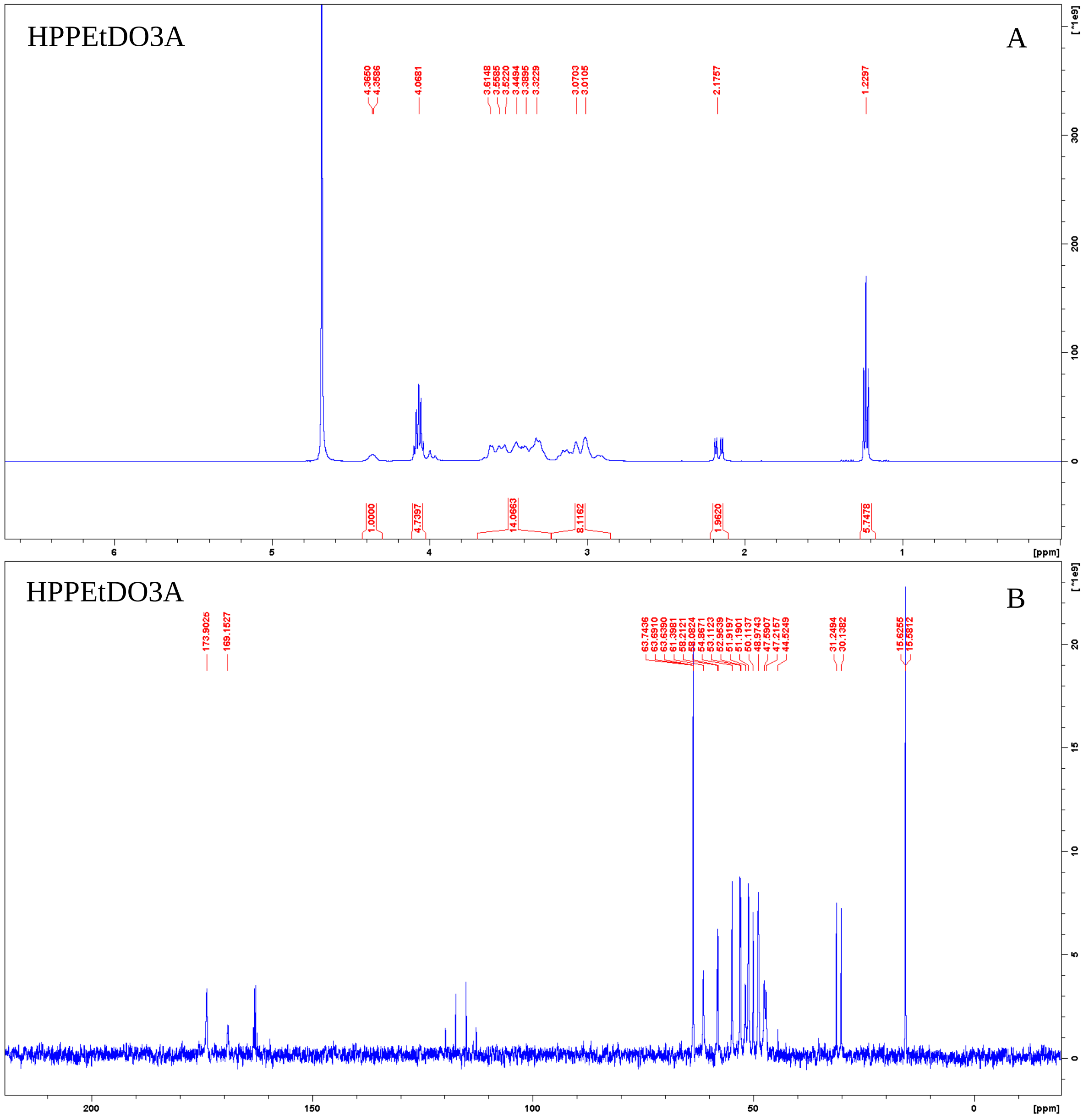

Figure S13. A: ^{1}H NMR spectrum of HPPEtDO3A; B: ^{13}C NMR spectrum of HPPEtDO3A.

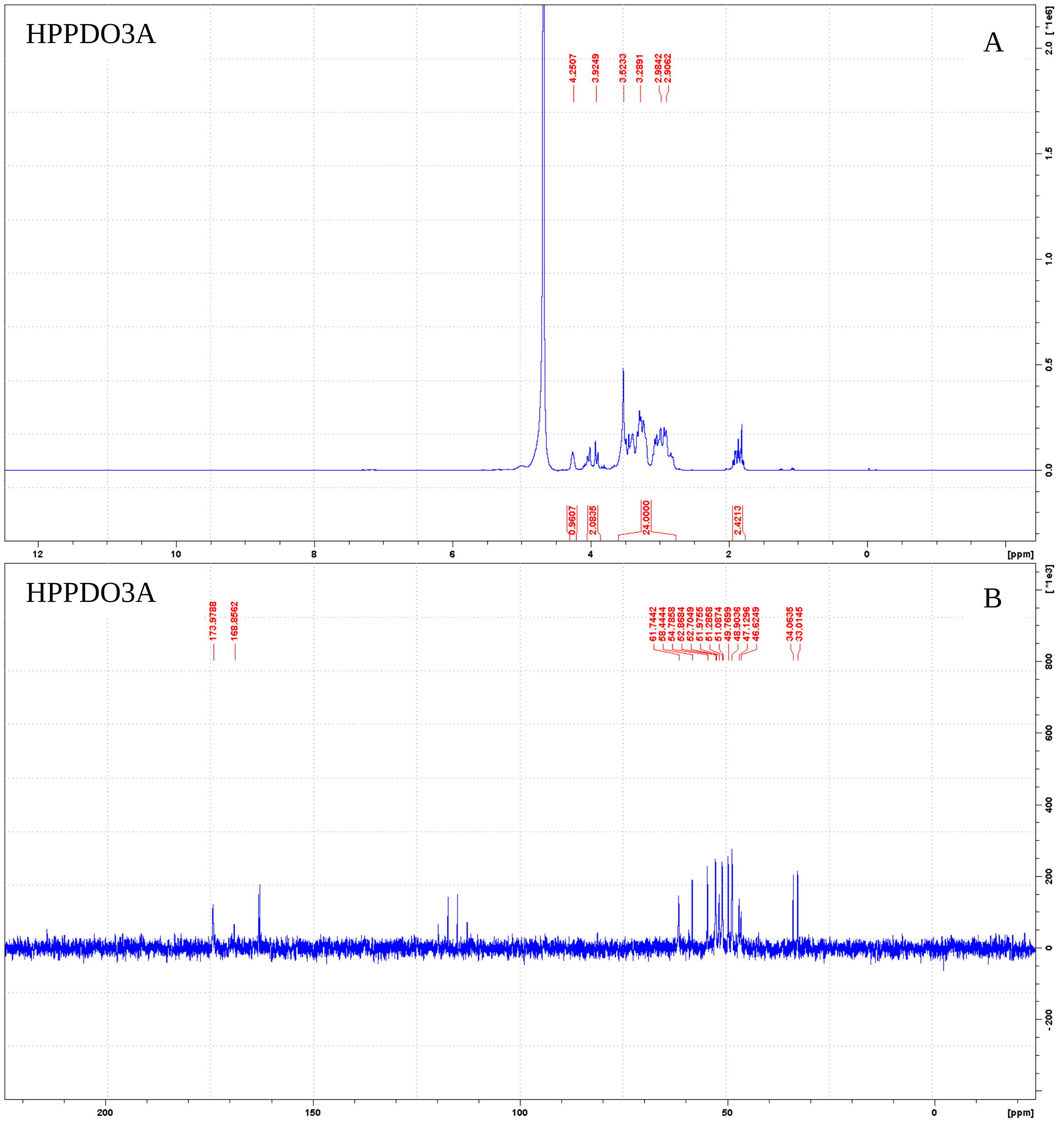

Figure S14. A: ^{1}H NMR spectrum of HPPDO3A; B: ^{13}C NMR spectrum of HPPDO3A.

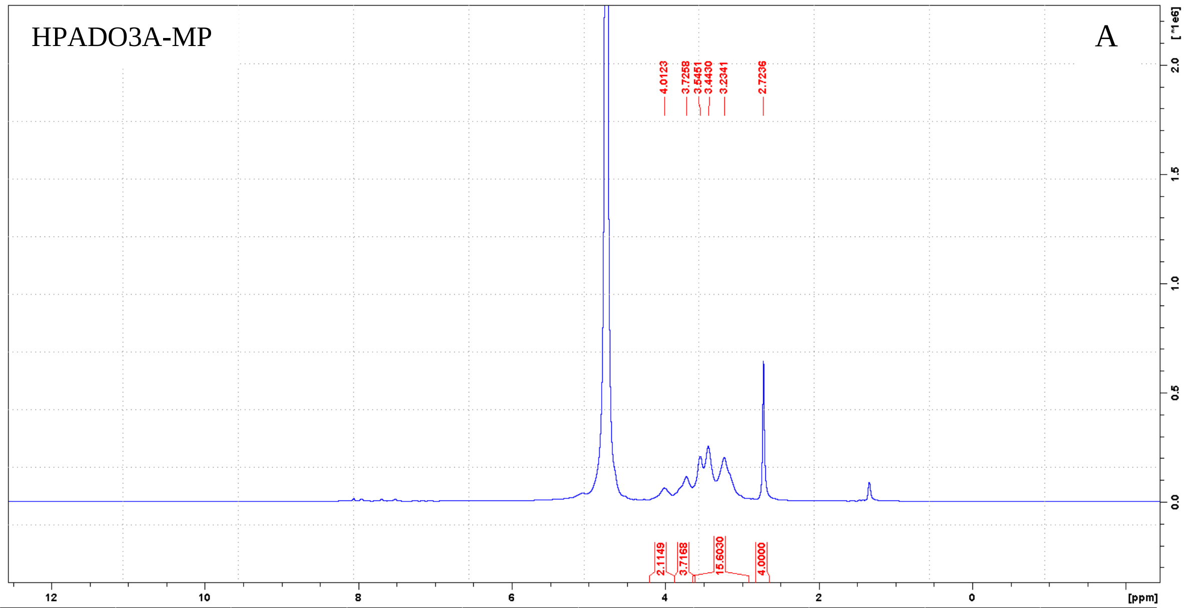

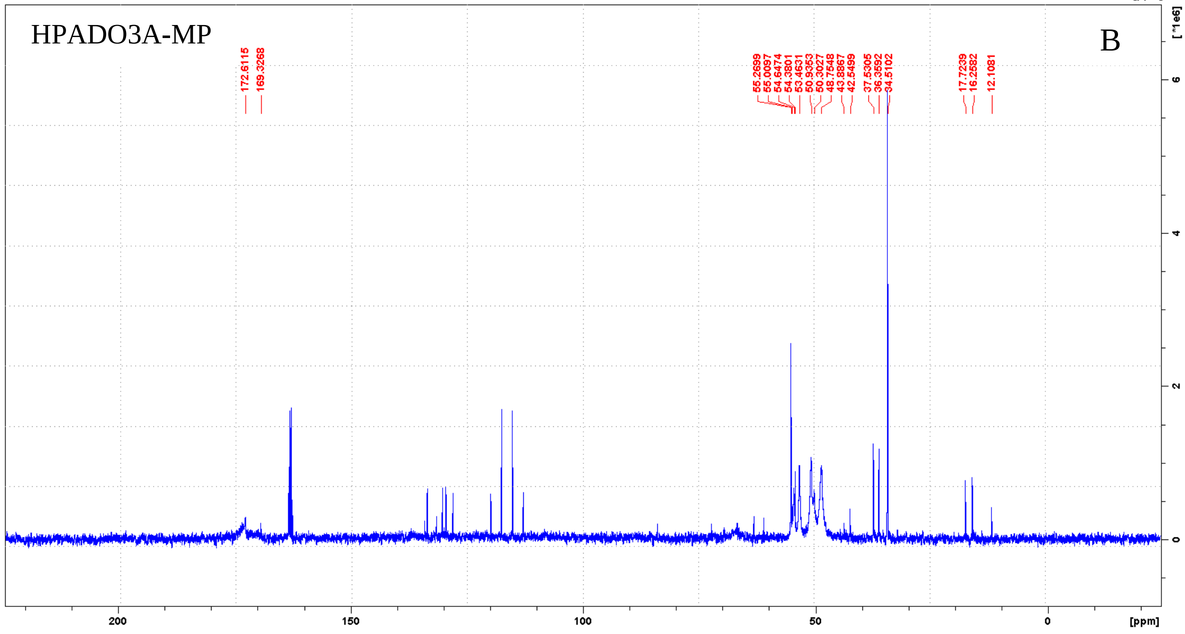

Figure S15. A: ^{1}H NMR spectrum of HPADO3A-MP; B: ^{13}C NMR spectrum of HPADO3A-MP.

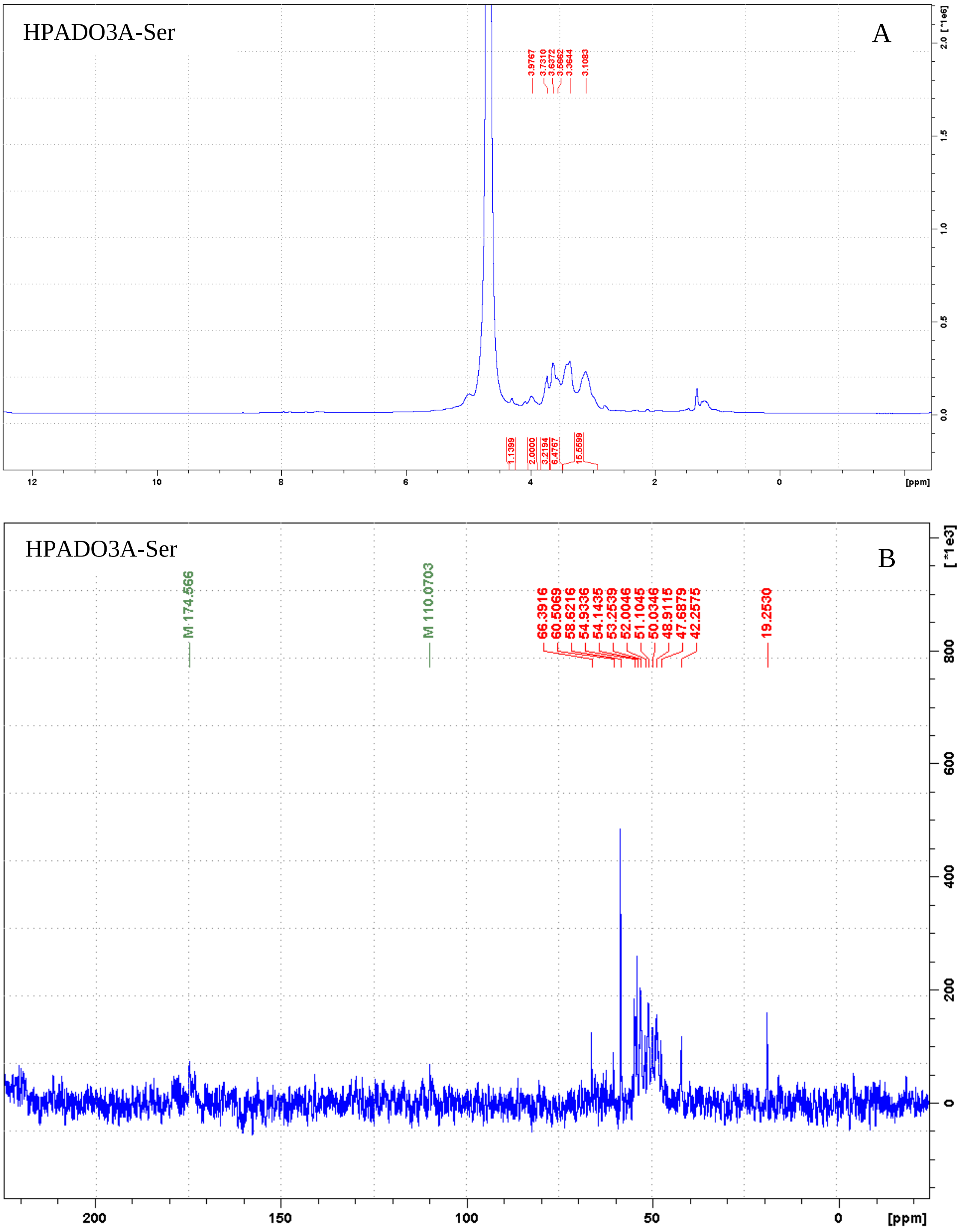

Figure S16. A: ^{1}H NMR spectrum of HPADO3A-Ser; B: ^{13}C NMR spectrum of HPADO3A-Ser.

References

([i]) Luz, Z.; Meiboom, S. *J. Chem. Phys.* **1964**, *40*, 2686.

([ii]) The Chemistry of Contrast Agents in Medical Magnetic Resonance Imaging (Eds: Merbach, A. E.; Tóth, É.), Wiley, New York, **2001**.

([iii]) Freed, J. H. *J. Chem. Phys.* **1978**, *68*, 4034.

([iv]) Koenig, S. H.; Brown III, R. D. *Prog. Nucl. Magn. Reson. Spectrosc.* **1991**, *22*, 487.
